# Alterations in regional homogeneity assessed by fMRI in patients with migraine without aura stratified by disease duration

**DOI:** 10.1186/1129-2377-14-85

**Published:** 2013-10-17

**Authors:** Ling Zhao, Jixin Liu, Xilin Dong, Yulin Peng, Kai Yuan, Fumei Wu, Jinbo Sun, Qiyong Gong, Wei Qin, Fanrong Liang

**Affiliations:** 1 Acupuncture and Tuina School, Chengdu University of Traditional Chinese Medicine, Chengdu, Sichuan 610075, China; 2 Life Sciences Research Center, School of Life Sciences and Technology, Xidian University, Xi’an, Shanxi 710071, China; 3 Department of Radiology, The Center for Medical Imaging, Huaxi MR Research Center, West China Hospital of Sichuan University, Chengdu, 610041 Sichuan, China

**Keywords:** Migraine without aura, Functional MRI, Regional homogeneity, Resting state, Disease duration

## Abstract

**Background:**

Advanced neuroimaging approaches have been employed to prove that migraine was a central nervous system disorder. This study aims to examine resting-state abnormalities in migraine without aura (MWoA) patients stratified by disease duration, and to explore the neuroimaging markers for reflecting the disease duration.

**Methods:**

40 eligible MWoA patients and 20 matched healthy volunteers were included in the study. Regional homogeneity (ReHo) analysis was used to identify the local features of spontaneous brain activity in MWoA patients stratified by disease duration, and analysis was performed to investigate the correlation of overlapped brain dysfunction in MWoA patients with different disease duration (long-term and short-term) and course of disease.

**Results:**

Compared with healthy controls, MWoA patients with long-term disease duration showed comprehensive neuronal dysfunction than patients with short-term disease duration. In addition, increased average ReHo values in the thalamus, brain stem, and temporal pole showed significantly positive correlations with the disease duration. On the contrary, ReHo values were negatively correlated with the duration of disease in the anterior cingulate cortex, insula, posterior cingulate cortex and superior occipital gyrus.

**Conclusions:**

Our findings of progressive brain damage in relation to increasing disease duration suggest that migraine without aura is a progressive central nervous disease, and the length of the disease duration was one of the key reasons to cause brain dysfunction in MwoA patients. The repeated migraine attacks over time result in resting-state abnormalities of selective brain regions belonging to the pain processing and cognition. We predict that these brain regions are sensitive neuroimaging markers for reflecting the disease duration of migraine patients without aura.

## Background

Migraine headache is a common neurological disorder which causes significant individual and societal burden due to pain and environmental sensitivities [1]. It was ranked the seventh highest among specific causes of disability globally. Migraine has two subtypes, and two thirds of migraine patients suffer from MWoA which is typically characterized as a unilateral and pulsating headache, and an autonomic nervous system dysfunction [2]. The recurrent headache manifests in attacks lasting 4–72 hours and affects patients 1–14 times each month in the episodic form. It is aggravated by routine physical activity, and is accompanied by vomiting, nausea, photophobia or phonophobia. Migraine may result in substantial pain, a decreased overall quality of life, and cause higher risks for ischemic stroke, unstable angina, and affective disorders than people without migraine [3-6].

Advanced neuroimaging approaches have been employed to investigate structural and functional brain changes in migraineurs, and proved that migraine was a central nervous system disorder [1]. The insula, anterior cingulate cortex (ACC), thalamus, prefrontal cortex (PFC), orbitofrontal cortex (OFC), parahippocampal cortex, periaqueductal gray matter (PAG), inferior frontal gyrus (IFG), brainstem, precentral gyrus, and cerebellum have been reported to show structural and functional alterations [7-15]. Furthermore, gray matter reduction based on voxel-based morphometric (VBM) studies was correlated with attack frequency or headache duration in migraine patients [13,16-18]. Moreover, task-related functional magnetic resonance imaging (fMRI) studies revealed abnormal activation of some brain regions associated with pain-related information processing in migraine patients, such as the ACC, the PFC, the OFC, insula and the supplementary motor area (SMA) [10,11,19]. Numerous findings have supported that migraine may have cumulative effects on brain structure and function, and repeated attacks over time would result in secondary damage on several brain regions involved in central pain processing [14,17,20-22]. Moreover, some preliminary neuroimaging studies provided some evidence about increased risk of brain abnormalities with increasing attack frequency [5,17,21,23] and disease duration [15,17,24] in migraineurs. However, few studies have evaluated the characteristic in the resting-state in MWoA patients stratified by disease duration.

In the current study, we performed a ReHo approach [25] to compare the blood oxygen level-dependent (BOLD) signals of the brains in MWoA patients along with healthy subjects during the resting-state. The ReHo method focuses on the similarities or coherence of the intraregional spontaneous low-frequency (<0.08 Hz) BOLD signal, which enables a novel perspective to understand the functional deficits in particular brain regions. An important advantage of using the ReHo method over other methods is that it can examine the regional activity characteristics of each voxel. It can also detect changes or modulations that are induced by different conditions across the whole brain in a voxel-by-voxel manner, without requiring any prior knowledge. Previously, our group has employed the ReHo method only to find that MWoA patients showed a significant decrease in ReHo values in the right ACC, PFC, OFC and SMA [12]. In addition, the ReHo values were negatively correlated with the duration of disease in the right ACC and PFC [12]. In order to further assess and validate whether some brain abnormalities serve as markers for disease history in MWoA patients, we investigated the resting-state difference between MWoA patients with long-term (LT) disease duration and MWoA patients with short-term (ST) disease duration. We hypothesized that, as compared with healthy controls, (1) MWoA patients with LT disease duration would display more neuronal dysfunction than patients with ST disease duration; (2) the overlapped brain dysfunction in LT and ST patients group may be associated with the course of disease in migraineurs.

## Methods

### Study participants

40 eligible MWoA patients were recruited from the neurology department of the Teaching Hospital of Chengdu University of Traditional Chinese Medicine. The diagnosis of MWoA was established according to the classification criteria of the International Headache Society (IHS) [26]. The inclusion criteria were as follows:(1) all subjects were right-handed, and had 2 to 8 migraine attacks per month during the last 3 months and during the baseline period (4 weeks before enrolment); (2) all subjects were 18 to 55 years of age; in addition, start of headache should be before the age of 50 years; (3) had received education for more than 6 years and had completed a baseline headache diary; (4) MWoA patients were selected on the basis of disease duration >10 years (LT) or < 5 years (ST); (5) had no migraine 72 hours prior to the scan; (6) no habit of long-term analgesics consumption; (7) did not take any prophylactic migraine medication during the previous month; and (8) no contraindications for exposure to a high magnetic field. Healthy subjects were recruited from the local community and were screened by a neurologist specialized in headaches. 20 right-handed, age-matched and education-matched healthy subjects were enrolled. They either had no headache days per year or had family members who suffered regularly from a migraine or other headache.

Exclusion criteria for MWoA patients and healthy controls were: (1) existence of neurological diseases; (2) had hypertension, diabetes mellitus, hypercholesteremia, vascular/heart disease, and major systemic conditions; (3) pregnant or lactating women; (4) alcohol or drug abuse; (5) any neuroimaging research study participation during the last 6 months; and (6) inability to understand the doctor’s instructions.

This study was approved by the ethics committee at the Teaching Hospital of Chengdu University of Traditional Chinese Medicine. All subjects gave written, informed consent after the experimental procedures had been fully explained.

### Study design

All patients should have recorded headache diaries for 4 weeks (baseline phase) before enrolment to assess disease activity (disease duration, headache degree, and attack frequency). Patients meeting the inclusion criteria were assigned to two groups based on different disease duration after the baseline period.

The headache diary documented the migraine attack frequency and severity of headache according to the guidelines of the IHS for clinical trials for migraine [27]. The VAS score 0–10 measured the intensity of headache. fMRI scans were scheduled 2 weeks after enrolment. In addition, records in the headache diary were checked to insure every patient did not suffer from a migraine attack at least 72 hours prior to the brain scan.

### Imaging data acquisition

The imaging data were carried out in a 3 Tesla Siemens MRI system (Allegra, Siemens Medical System, Erlangen, Germany) at the Huaxi MR Research Center, West China Hospital of Sichuan University, Chengdu, China. A standard eight-channel phase-array head coil was used, along with restraining foam pads to minimize head motion and to diminish scanner noise. Prior to the functional run, a high-resolution T1structural image for each subject was acquired using a three-dimensional MRI sequence with a voxel size of 1 mm^3^ employing an axial fast spoiled gradient recalled sequence (TR = 1900 ms, TE = 2.26 ms, data matrix = 256 × 256, flip angle = 9°, FOV = 256 mm × 256 mm). The structural images were examined to exclude the possibility of clinically silent lesions for all of the participants by two expert radiologists. The resting-state functional images were obtained with echo-planar imaging (EPI) (30 continuous slices with a slice thickness = 5 mm, TR = 2000 ms, TE = 30 ms, flip angle = 90°, FOV = 240 mm × 240 mm, matrix = 64 × 64). During 6-min fMRI scanning, participants were instructed to keep their eyes closed, relax, move as little as possible, and stay awake. It needs to be emphasized that if there was an attack for migraine patients in the check reservation, they could not be scanned and the scan would be postponed to ensure they were scanned during the migraine interval.

### Data preprocessing

In the functional image data preprocessing, the first five scans were discarded to eliminate nonequilibrium effects of magnetization and to allow participants to become familiar with the scanning circumstances. Data preprocessing was done using Statistical Parametric Mapping (SPM5, http://www.fil.ion.ucl.ac.uk/spm). The images were corrected for the acquisition delay between slices, aligned to the first image of each session for motion correction and spatially normalized to the standard Montreal Neurological Institute (MNI) template in SPM5. We calculated the maximum excursion movement values for each of the translation planes (x, y, and z) and each of the rotation planes (roll, pitch, and yaw) for every participant. None of them had head movements exceeding 1 mm on any axis and head rotation greater than 1° during the entire fMRI scan. Finally, a band-pass filter (0.01 Hz < f < 0.08 Hz) was applied to remove physiological and high-frequency noise.

### Data analysis

Baseline and demographic data were analyzed by SPSS 14.0 statistical software (SPSS Inc., Chicago, IL, USA). Baseline characteristics were summarized by descriptive statistics for each group and in the total study population. Two independent-sample *t*-tests were used to examine differences between groups (95% CI, 2-sided).

Kendall’s coefficient of concordance (KCC) [28] was used to evaluate ReHo [25], which was performed using the Resting-State fMRI Data Analysis Toolkit (X.-W. Song et al., Beijing Normal University, Beijing, China, http://www.restfmri.net). Individual ReHo maps were generated by assigning each voxel a value corresponding to the KCC of its time series with its nearest 26 neighboring voxels [25]. Then, a mask (made from the MNI template to assure matching with the normalization step) was used to remove non-brain tissues and noise from the ReHo maps. Only the voxels within the mask were analyzed further. The individual ReHo maps were standardized by their own mean KCC within the mask. Then, a Gaussian kernel with a full-width at half-maximum of 4 mm was used to smooth the images in order to reduce noise and residual differences. Controlling for age, two independent-sample *t*-tests were used to compare the ReHo results between different groups. The false discovery rate (FDR) was used to correct the multiple comparisons. In addition, correlation analyses were performed in order to delineate possible correlations between average ReHo values of the overlapped brain dysfunctional regions in LT and ST groups and the disease duration.

## Results

### Participants

There were no significant differences in the demographics including age, gender, and education between MWoA patients and healthy subjects (*p >* 0.05) (Table 1). There were no significant differences in the demographics including sex, education, family history, migraine attack frequency, and visual analogue scale (VAS) score between ST group patients and LT group subjects (*p >* 0.05). Patients in the LT group were older and had longer disease duration compared with patients in the ST group (*p <* 0.05) (Table 2).

**Table 1:** Baseline and demographics for MWoA patients and healthy subjects

**Items**	**MWoA patients (n = 40)**	**Healthy subjects (n = 20)**	***P*value**
Age (years) mean (SD)	30.5(10.8)	28.4(8.9)	0.4569
Gender (male/female)	12/28	5/15	0.4658
Education (years)	12.6(4.7)	14.2(6.4)	0.2764
Mean disease duration in years (SD)	10.15(7.01)	NA	

Notes: SD, Standard deviation.

**Table 2:** Baseline and demographics for MWoA patients

**Items**	**ST group (n = 20)**	**LT group (n = 20)**	***P *value**
Age (years) mean (SD)	27.12 (8.18)	37.52 (12.2)	0.0009
Gender (male/female)	5/15	7/13	0.7311
Education (years)	13.2 (6.03)	13.8 (5.06)	0.7352
Family history (Y/N)	6/14	8/12	0.3705
Disease duration in years (SD)	4.05 (1.64)	16.25 (1.47)	0.0000
Affect frequency per month^*^ (SD)	4.5 (3.5)	5.38 (5.8)	0.5647
VAS score^*^	5.37 (1.34)	5.0 (1.8)	0.4654

Notes: SD, Standard deviation; VAS, Visual Analogue Scale; Y, yes; N, no.

^*^measured for the 4 weeks before enrolment.

### Neuroimaging results

Compared with healthy subjects, MWoA patients with ST disease duration showed significantly higher ReHo values in the bilateral thalamus, IFG (Brodmman area (BA) 47), middle occipital gyrus (MOG) (BA19), left insula (BA13), caudate, middle frontal gyrus (MFG) (BA8), middle temporal gyrus (MTG) (BA37), inferior occipital gyrus (IOG) (BA19), right ACC (BA32), medial frontal gyrus (MeFG) (BA25), and superior temporal gyrus (STG) (BA42). The results revealed MWoA patients with ST disease duration showed a significant decrease in ReHo values in the bilateral MFG (BA8,BA10), MTG (BA21), left lingual gyrus (BA17), right MOG (BA19), cerebellum, and brain stem (controlling for age, *p* <0.01, FDR corrected) (Additional file 1, Figure 1a).

**Figure 1:**
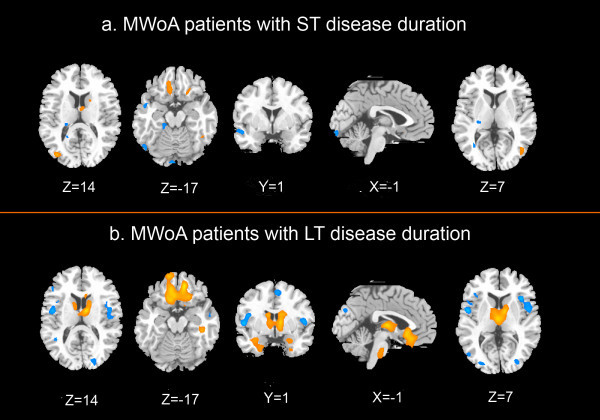
**Differences between MWoA patients and healthy subjects in ReHo values. a**. MWoA patients with ST disease duration; **b**. MWoA patients with LT disease duration; *p* < 0.01, FDR corrected; Warm colors indicate ReHo increases in MWoA patients; cool colors indicate ReHo decreases in MWoA patients.

In this study, the MWoA patients with LT disease duration showed increased ReHo values in the bilateral ACC (BA24, BA32), amygdala, thalamus, caudate, lentiform nucleus, uncus, IFG (BA11, BA47), MFG (BA11), SFG (BA6, BA11), MTG (BA21), temporal pole (BA38), cerebellum, brain stem (including pons, medulla, and midbrain), and left hippocampus compared with healthy subjects. On the contrary, the results seemed decreased in the bilateral ACC (BA24), insula (BA13), IFG (BA45, BA47), MFG (BA6), MeFG (BA6, BA8), SFG (BA6), MTG (BA21, BA39), MOG (BA18, BA19), cuneus (BA18, BA19), lingual gyrus (BA18, BA19), inferior parietal lobule (IPL) (BA40), postcentral gyrus (BA6, BA43), and precuneus (BA19, BA31), left fusiform gyrus (BA19), and right posterior cingulate cortex (PCC) (BA31) (controlling for age, *p* < 0.01, FDR corrected) (Additional file 2, Figure 1b).

**Figure 2:**
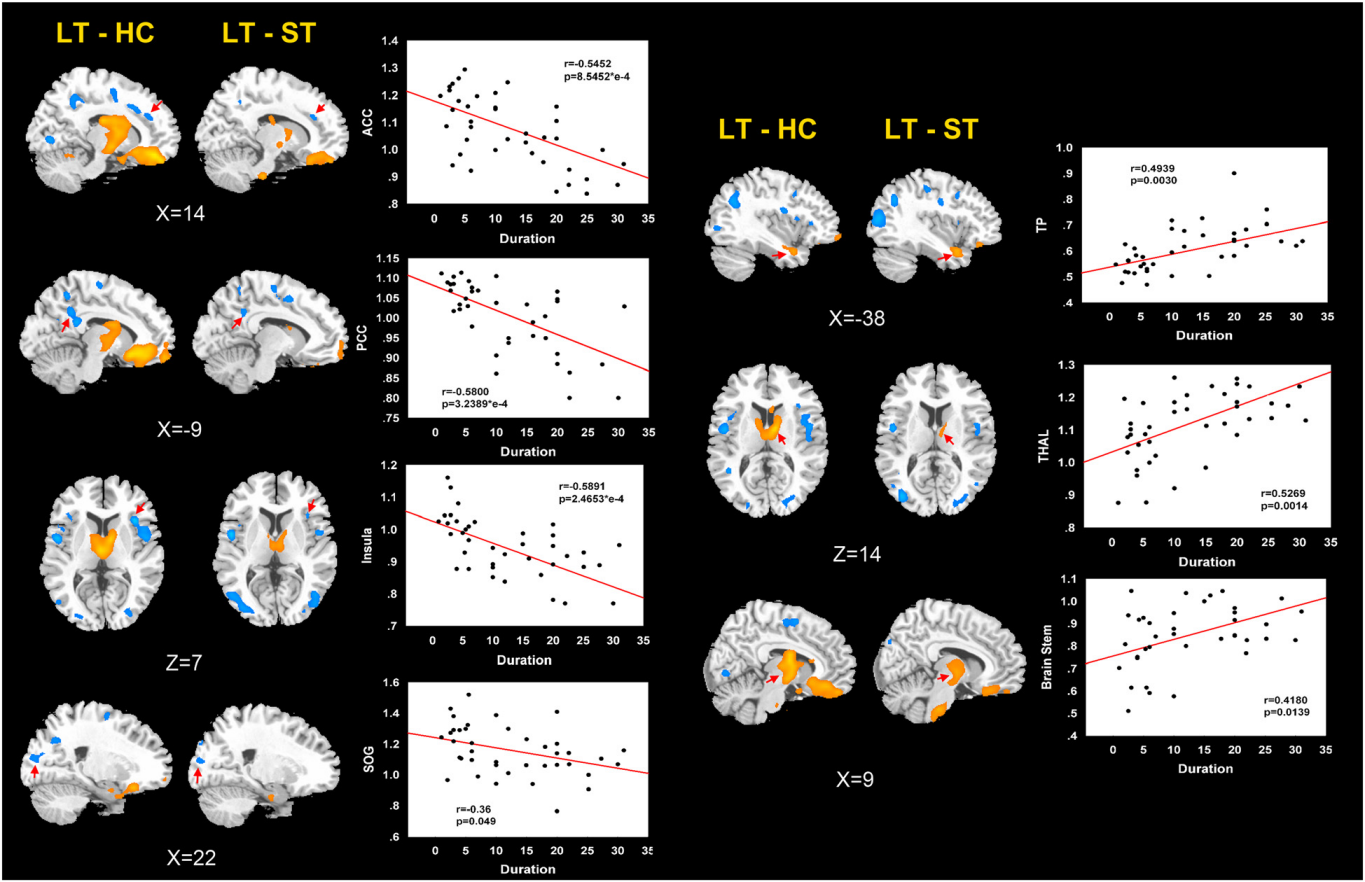
**The correlation of average ReHo values of the overlapped brain dysfunction in LT *vs *HC and LT *vs *ST with the disease duration.** Warm colors indicate ReHo increases in MWoA patients; cool colors indicate ReHo decreases in MWoA patients; ACC, anterior cingulate cortex; THAL, thalamus; TP, temporal pole; PCC, posterior cingulate cortex; SOG, superior occipital gyrus; LT, MWoA patients with long-term disease duration; ST, MWoA patients with short-term disease duration; HC, healthy controls.

Correlation analysis results demonstrated that increased average ReHo values in the thalamus (r = 0.5269, *p* = 0.0014), brain stem (r = 0.4180, *p* = 0.0139), and temporal pole (r = 0.4939, *p* = 0.0030) showed significantly positive correlations with the disease duration (Figure 2). There were respectively significant negative correlations between the decreased average ReHo values of the ACC (r = -0.5452, *p* = 8.5452*e-4), insula (r = -0.5891, *p* = 2.4653*e-4), PCC (r = -0.5800, *p* = 3.2389*e-4), SOG (r = -0.36, *p* = 0.049) and the disease duration (Figure 2). The correlation between VAS score and attack frequency and resting-state properties were also checked, but no results exceeded the threshold.

## Discussion

To our knowledge, this study is the first one to investigate characteristic of regional homogeneity in patients with episodic migraine without aura stratified by disease duration. ReHo hypothesizes that a given voxel is temporally similar to that of its neighbors [25]. It is calculated by using Kendall’s coefficient of concordance, which could obtain reliable results in a resting-state fMRI data analysis [29]. Therefore, ReHo reflects the temporal homogeneity of the regional BOLD signal rather than its density. Compared with healthy controls, several common brain regions showed abnormalities in MWoA patients with ST and LT disease duration during the resting-state, including IFG, MFG, MTG, ACC, thalamus, and basal ganglia. These results were mainly involved in pain-related processing, and were similar to previous reports in migraineur studies which focused on structural [16,17,30,31], task-related [11,19,21], and resting-state [12-15,24] abnormalities. Furthermore, compared with healthy controls, MWoA patients with LT disease duration might display comprehensive neuronal dysfunction than patients with ST disease duration. PCC, lentiform nucleus, uncus, temporal pole, MOG, cuneus, fusiform gyrus, inferior parietal lobule, postcentral gyrus, precuneus, and brain stem were only found in MWoA patients with LT disease duration. In the current study, abnormal ReHo in MwoA patients was relevant to the changes of temporal aspects of neural activity in the brain regions. Increased or decreased ReHo suggests that neural function in local regions is more or less synchronous during resting-state. The results demonstrated that the long history of disease might contribute to accumulating brain damage due to the repetitive occurrence of pain-related processes.

We were interested in whether brain abnormalities would progressively influence individuals as the result of migraine attack history. To explore which brain regions might relate to the course of disease, a correlation analysis was performed. The results showed that the average ReHo value of the thalamus, brain stem, and temporal pole were positively related to the disease duration. The ReHo value of the ACC, insula, PCC and SOG were negatively correlated with the history of MWoA. Therefore, the ReHo increase in the thalamus, brain stem, and temporal pole in MwoA patients may reflect a dynamic compensation for the disorder signals from the brain, whereas the decreased hemodynamic synchronization in the ACC, insula, PCC, and SOG could be explained by MwoA -related dysfunction. Additionally, we speculated these ReHo changes might reflect not only as a consequence of repeated painful attacks in a pain disorder, but also as indicators specific to migraine without aura.

As we all know, the ACC, insula, and thalamus are the key regions composed of the “pain matrix”. Recent neuroimaging evidence supported that the ACC and insula were the common “brain signature” structures in chronic pain diseases, such as fibromyalgia [32], irritable bowel syndrome [33], chronic tension type headache [34], and migraine [16,35,36]. ACC has a close interconnection with the insula, thalamus, prefrontal cortex, and other subcortical structures, and is considered to be implicated in both affective and cognitive-attentional dimensions of pain and plays a deterministic role in pain modulation and analgesia [37]. In the current study, ACC demonstrated negative correlation with disease duration, which was consistent with previous correlation analysis reports separately on regional metabolism [35] and average ReHo values [12] of the ACC. The insula is a complex, multisensory integration area that is involved in processing many aspects involved with the conscious experience of pain such as affect, autonomic activity and interoception. A recent study strongly suggested that if the full pain experience involves the pain matrix network, a part of the insula seems to play a leading role in the triggering of this network and the resulting emergence of the subjective pain experience [38]. Functional imaging experiments have revealed that the insula is a major site for emotional processing, and it also processes sensory-discriminative aspects of pain perception [39]. Coghill et al., reported the insula cortex plays reciprocal role in pain, emotions and pain-related emotions, due to its anatomic connections [40]. We found that the progressive dysfunction of the insula showed a significant correlation with disease history, and did not detect a significant relationship between the insula and headache degree or attack frequency. The thalamus was also found to have a dysfunction in migraine patients in previous documents [41-43], but few studies have evaluated the correlation between the abnormality of the thalamus and clinical parameters. The thalamus is the “relay center” of the brain, and it is involved in the formation of the lateral and medial pain system. The lateral nuclei of the thalamus deal with discriminative sensory pain transmission, and the medial nuclei of the thalamus are involved in emotional and somatic responses to pain [44]. In the current study, increased ReHo values of the thalamus were positively correlated with disease duration, suggesting that this cumulative alteration was mainly due to migraine, and not only the secondary effect of having migraine headaches. Our results demonstrated that the ACC, insula, and thalamus were not only related to central pain processing for migraine without aura, but also involved in expressing the relationship between brain dysfunction and disease history.

Moreover, several independent functional imaging studies have reinforced the fact that the pathogenesis of migraine is related to the dysfunction of the brain stem. A series of positron emission tomographic (PET) studies consistently observed an increase in regional cerebral blood flow in the brain stem during migraine attacks [41,45-47], and the brain stem was also found to be activated in migraine patients with some stimulus detected by fMRI [48-50]. Dysfunction of the brain stem is involved in anti­nociception, extracerebral and intracerebral vascular control and sensory gating provides an explanation for many of the facets of migraine. In this study, increased ReHo values in the brain stem were related with disease duration during the resting-state facilitation that the brain stem has a crucial role in migraine, and may serve as an indicator to reflect the progress of migraine.

PCC participates in the composition of the default mode network (DMN), and it seldom detected significant abnormal findings in migraine patients checked by neuroimaging. It is not the traditional pain-processing area, but recently, Loggia et al. reported that some DMN subregions (such as the PCC) respond in a perception-related manner to pain, suggesting closer linkage between the DMN and pain processing than previously thought [51]. Furthermore, the PCC is recognized that subjects with cognitive impairment showed reduced cerebral blood flow in the PCC, and some clinical evidence indicated that migraine patients had deficits in cognitive function relative to healthy controls [52-54]. Our findings of progressive ReHo changes in the PCC in relation to increasing disease duration suggest that repeated migraine attacks over time may lead to resting-state abnormalities of selective brain regions belonging to pain perception and cognitive control. The temporal pole was found to have an increase in the fMRI BOLD response during the interictal period in migraineurs in response to a thermal stimulus [11], and also showed significantly higher activation during odor stimulation by H_2_^15^O-PET [55]. The role of the temporal pole in pain processing is not well understood, but it is an associative multisensory area and plays a role in assigning affective tone to short-term memories relating to pain, which may be related to reports of impaired memory in migraine patients during the interictal period [11]. We found the ReHo properties of the temporal pole were positively correlated with the duration of disease, which suggests that temporal pole excitability as sensitization during both the resting-state and stimulation may contribute to repeated migraine attack. Lesions in the occipital lobe result in visual disturbance, memory deficits and motion perception disorders. Occipital lobes had bilateral hypoperfusion in a patient with spontaneous migraine without aura as detected by PET [56,57], and an fMRI study found that the occipital cortex showed structural deficits in MWoA patients [13]. In the current study, our results showed decreased ReHo values in the SOG which were negatively correlated with the disease duration. Therefore, we inferred that the observed PCC, temporal pole and SOG dysfunction in MWoA patients may provide a potential neurobiological mechanism for cognitive deficits in migraineurs.

There are some limitations in the present study. Firstly, disease duration was used to classify the MWoA patients with ST and LT, not including the MWoA patients with moderate-term disease duration (between 5 years and 10 years). Further studies need to recruit a large number of MWoA patients and stratify the detailed data, and give more evidence to strengthen our findings. Secondly, in order to test the reproducibility of our results and to verify the consequences of brain damage in migraineurs, further neuroimaging investigations have to quantify brain abnormalities in a longitudinal design. Lastly, we will plan to assess cerebral structural changes in MwoA patients by using DTI, VBM, or surface-based techniques in the future work, and help us to better understand the pathophysiology of migraine.

## Conclusion

In conclusion, the current study employed the ReHo method to investigate the difference in resting-state properties between MWoA patients stratified with different disease duration and healthy controls, as well as the correlation of abnormal cerebral activity in MWoA patients and disease duration. Our findings of progressive abnormal ReHo values in relation to increasing disease duration suggest that migraine without aura is a progressive central nervous disease, and the length of the disease duration was one of the key reasons to cause brain dysfunction in MwoA patients. The repeated migraine attacks over time result in resting-state abnormalities of selective brain regions belonging to the pain processing and cognition. Our results provided more scientific and sensitive neuroimaging markers for reflecting the disease duration of migraine patients without aura, and helped to identify indicators of predilection sites for possible progressive brain damage in migraineurs. It is expected that these findings may be advance the understanding of the pathology of migraine without aura and helpful to the diagnosis and therapy for MwoA patients. For example, take the appropriate individual treatment program depending on the different length of duration of disease, and increase some special assessment in brain function for migraine patients with long disease duration.

## Abbreviations

ACC: Anterior cingulate cortex; PFC: Prefrontal cortex; OFC: Orbitofrontal cortex; PAG,: Periaqueductal gray matter; IFG: Inferior frontal gyrus; VBM: Voxel-based morphometric; fMRI: Functional MRI; SMA: Supplementary motor area; ReHo: Regional homogeneity; BOLD: Blood oxygen level-dependent; IHS: International Headache Society; VAS: Visual analogue scale; KCC: Kendall’s coefficient of concordance; FDR: False discovery rate; MOG: Middle occipital gyrus; MFG: Middle frontal gyrus; MTG: Middle temporal gyrus; IOG: Inferior occipital gyrus; SOG: Superior occipital gyrus; MeFG: Medial frontal gyrus; STG: Superior temporal gyrus; DMN: Default mode network.

## Competing interests

The authors declare that they have no conflicts of interest or financial disclosures.

## Supplementary Material

Additioanl file 1 Comparison between MWoA patients with ST disease duration and healthy controls. Click here for file

Additional file 2 Comparison between MWoA patients with LT disease duration and healthy controls. Click here for file
