# Supplementary material for: Alterations in regional homogeneity assessed by fMRI in patients with migraine without aura stratified by disease duration
Source: J Headache Pain. 2013 Oct 17;14(1):85. doi: 10.1186/1129-2377-14-85 (PMC3853130; doi:10.1186/1129-2377-14-85)
Supplement: Additioanl file 1 — Comparison between MWoA patients with ST disease duration and healthy controls. [file 1129-2377-14-85-S1.doc]

**Additioanl file 1** **Comparison between** **MWoA patients with ST disease duration and healthy control**

| Region | BA | Hemi | MWoA Patients > Healthy Controls | | | | MWoA Patients < Healthy Controls | | | | |
| --- | --- | --- | --- | --- | --- | --- | --- | --- | --- | --- | --- |
|  |  |  | Talairach | | | *t-value* |  | Talairach | | | *t-value* |
|  |  |  | x | y | z |  |  | x | y | z |  |
| **Limbic System** |  |  |  |  |  |  |  |  |  |  |  |
| Anterior Cingulate Cortex | 32 | L | - | - | - | - |  | - | - | - | - |
|  |  | R | 6 | 20 | -9 | 2.71 |  | - | - | - | - |
| Thalamus | - | L | -6 | -5 | 11 | 2.72 |  | - | - | - | - |
|  |  | R | 15 | -14 | 17 | 3.09 |  | - | - | - | - |
| Insula | 13 | L | -48 | -34 | 21 | 3.13 |  | - | - | - | - |
|  |  | R | - | - | - | - |  | - | - | - | - |
| Caudate | - | L | -9 | -2 | 17 | 3.14 |  | - | - | - | - |
|  |  | R | - | - | - | - |  | - | - | - | - |
| **Frontal Cortex** |  |  |  |  |  |  |  |  |  |  |  |
| Inferior Frontal Gyrus | 47 | L | -21 | 19 | -19 | 3.07 |  | - | - | - | - |
|  |  | R | 15 | 31 | -17 | 3.06 |  | - | - | - | - |
| Middle Frontal Gyrus | 8/10 | L | -33 | 29 | 46 | 3.05 |  | -30 | 59 | 22 | -2.75 |
|  |  | R | - | - | - | - |  | 24 | 17 | 46 | -2.76 |
| Medial Frontal Gyrus | 25 | L | - | - | - | - |  | - | - | - | - |
|  |  | R | 12 | 28 | -17 | 3.30 |  | - | - | - | - |
| **Temporal Cortex** |  |  |  |  |  |  |  |  |  |  |  |
| Middle Temporal Gyrus | 37/21 | L | -45 | -67 | 6 | 3.31 |  | -59 | 2 | -18 | -2.95 |
|  |  | R | - | - | - | - |  | 56 | -1 | -10 | -2.76 |
| Superior Temporal Gyrus | 42 | L | - | - | - | - |  | - | - | - | - |
|  |  | R | 68 | -14 | 6 | 2.83 |  | - | - | - | - |
| **Occipital Cortex** |  |  |  |  |  |  |  |  |  |  |  |
| Inferior Occipital Gyrus | 19 | L | -42 | -73 | -4 | 3.08 |  | - | - | - | - |
|  |  | R | - | - | - | - |  | - | - | - | - |
| Middle Occipital Gyrus | 19 | L | -45 | -70 | 6 | 3.16 |  | - | - | - | - |
|  |  | R | 42 | -78 | 12 | 2.99 |  | 53 | -67 | -9 | -2.77 |
| Lingual Gyrus | 17 | L | - | - | - | - |  | 9 | -94 | -13 | -2.80 |
|  |  | R | - | - | - | - |  | - | - | - | - |
| **Cerebellum** |  |  |  |  |  |  |  |  |  |  |  |
| Declive | - | L | - | - | - | - |  | - | - | - | - |
|  |  | R | - | - | - | - |  | 30 | -59 | -15 | -2.72 |
| Culmen | - | L | - | - | - | - |  | - | - | - | - |
|  |  | R | - | - | - | - |  | 18 | -30 | -21 | -2.71 |
| **Brain Stem** |  |  |  |  |  |  |  |  |  |  |  |
| Pons | - | L | - | - | - | - |  | - | - | - | - |
|  |  | R | - | - | - | - |  | 18 | -27 | -19 | -2.74 |

Note: *p*<0.01 (FDR, Corrected); R: right; L: left; Hemi: hemisphere
