# Supplementary material for: Alterations in regional homogeneity assessed by fMRI in patients with migraine without aura stratified by disease duration
Source: J Headache Pain. 2013 Oct 17;14(1):85. doi: 10.1186/1129-2377-14-85 (PMC3853130; doi:10.1186/1129-2377-14-85)
Supplement: Additional file 2 — Comparison between MWoA patients with LT disease duration and healthy controls. [file 1129-2377-14-85-S2.doc]

**Additional file 2 Comparison between** **MWoA patients with LT disease duration and healthy controls**

| Region | BA | Hemi | MWoA Patients > Healthy Controls | | | | MWoA Patients < Healthy Controls | | | | |
| --- | --- | --- | --- | --- | --- | --- | --- | --- | --- | --- | --- |
|  |  |  | Talairach | | | *t-value* |  | Talairach | | | *t-value* |
|  |  |  | x | y | z |  |  | x | y | z |  |
| **Limbic System** |  |  |  |  |  |  |  |  |  |  |  |
| Anterior Cingulate Cortex | 24/32 | L | -3 | 20 | -9 | 3.90 |  | -3 | -3 | 47 | -2.72 |
|  |  | R | 6 | 20 | -6 | 5.01 |  | 12 | -1 | 47 | -2.73 |
| Posterior Cingulate Cortex | 31 | L | - | - | - | - |  | - | - | - | - |
|  |  | R | - | - | - | - |  | 12 | -42 | 35 | -2.73 |
| Hippocampus | - | L | -27 | -10 | -22 | 3.50 |  | - | - | - | - |
|  |  | R | - | - | - | - |  | - | - | - | - |
| Amygdala | - | L | -24 | -4 | -17 | 2.94 |  | - | - | - | - |
|  |  | R | 30 | -4 | -17 | 3.40 |  | - | - | - | - |
| Thalamus | - | L | -9 | -2 | 8 | 5.16 |  | - | - | - | - |
|  |  | R | 3 | -14 | 6 | 5.13 |  | - | - | - | - |
| Insula | 13 | L | - | - | - | - |  | -45 | -20 | 15 | -2.7 |
|  |  | R | - | - | - | - |  | 39 | 9 | 8 | -2.72 |
| Caudate | - | L | -9 | -2 | 17 | 4.74 |  | - | - | - | - |
|  |  | R | 12 | 1 | 17 | 4.15 |  | - | - | - | - |
| Lentiform Nucleus | - | L | -12 | -3 | 0 | 3.74 |  | - | - | - | - |
|  |  | R | 12 | -3 | 0 | 3.17 |  | - | - | - | - |
| Uncus | 28 | L | -27 | -10 | -27 | 3.79 |  | - | - | - | - |
|  |  | R | 24 | 7 | -28 | 3.41 |  | - | - | - | - |
| **Frontal Cortex** |  |  |  |  |  |  |  |  |  |  |  |
| Inferior Frontal Gyrus | 11/45/47 | L | -12 | 37 | -20 | 5.12 |  | -33 | 24 | 4 | -3.08 |
|  |  | R | 15 | 20 | -16 | 4.87 |  | 39 | 23 | 2 | -2.89 |
| Middle Frontal Gyrus | 6/11 | L | -18 | 40 | -22 | 3.25 |  | -21 | 0 | 58 | -2.72 |
|  |  | R | 36 | 58 | -13 | 3.80 |  | 24 | -12 | 53 | -2.74 |
| Medial Frontal Gyrus | 6/8 | L | - | - | - | - |  | -9 | 6 | 52 | -2.78 |
|  |  | R | - | - | - | - |  | 6 | 51 | 39 | -2.72 |
| Superior Frontal Gyrus | 6/11 | L | -3 | 20 | 60 | 3.01 |  | -3 | 5 | 49 | -2.82 |
|  |  | R | 15 | 54 | -18 | 4.44 |  | 21 | 15 | 60 | -2.72 |
| **Temporal Cortex** |  |  |  |  |  |  |  |  |  |  |  |
| Middle Temporal Gyrus | 21/39 | L | -56 | -24 | -11 | 2.84 |  | -53 | -7 | -17 | -2.73 |
|  |  | R | 68 | -29 | -1 | 2.77 |  | 42 | -60 | 28 | -2.73 |
| Temporal Pole | 38 | L | -33 | 5 | -20 | 2.81 |  | - | - | - | - |
|  |  | R | 33 | 5 | -20 | 3.88 |  | - | - | - | - |
| **Occipital Cortex** |  |  |  |  |  |  |  |  |  |  |  |
| Inferior Occipital Gyrus | 19 | L | - | - | - | - |  | - | - | - | - |
|  |  | R | - | - | - | - |  | - | - | - | - |
| Middle Occipital Gyrus | 18/19 | L | - | - | - | - |  | -30 | -87 | 4 | -2.91 |
|  |  | R | - | - | - | - |  | 36 | -84 | 7 | -2.7 |
| Cuneus | 18/19 | L | - | - | - | - |  | -21 | -75 | 20 | -2.87 |
|  |  | R | - | - | - | - |  | 6 | -80 | 32 | -2.74 |
| Fusiform Gyrus | 19 | L | - | - | - | - |  | -21 | -75 | 20 | -2.87 |
|  |  | R | - | - | - | - |  | - | - | - | - |
| Lingual Gyrus | 18/19 | L | - | - | - | - |  | -9 | -73 | -1 | -2.75 |
|  |  | R | - | - | - | - |  | 18 | -67 | 1 | -2.73 |
| **Parietal Lobe** |  |  |  |  |  |  |  |  |  |  |  |
| Inferior Parietal Lobule | 40 | L | - | - | - | - |  | -48 | -53 | 44 | -2.74 |
|  |  | R | - | - | - | - |  | 39 | -53 | 39 | -2.99 |
| Postcentral Gyrus | 6/43 | L | - | - | - | - |  | -50 | -17 | 15 | -2.71 |
|  |  | R | - | - | - | - |  | 50 | -17 | 17 | -2.93 |
| Precuneus | 19/31 | L | - | - | - | - |  | -30 | -71 | 34 | -2.7 |
|  |  | R | - | - | - | - |  | 9 | -48 | 33 | -2.72 |
| **Cerebellum** |  |  |  |  |  |  |  |  |  |  |  |
| Declive | - | L | -39 | -65 | -19 | 2.96 |  | - | - | - | - |
|  |  | R | 30 | -74 | -19 | 3.05 |  | - | - | - | - |
| **Brain Stem** |  |  |  |  |  |  |  |  |  |  |  |
| Pons | - | L | 0 | -25 | -32 | 3.03 |  | - | - | - | - |
|  |  | R | 3 | -25 | -29 | 3.39 |  | - | - | - | - |
| Medulla | - | L | 0 | -28 | -36 | 3.15 |  | - | - | - | - |
|  |  | R | 3 | -25 | -37 | 3.17 |  | - | - | - | - |
| Midbrain | - | L | -9 | -9 | -7 | 4.85 |  | - | - | - | - |
|  |  | R | 9 | -9 | -7 | 4.22 |  | - | - | - | - |

Note: *p*<0.01 (FDR, Corrected); R: right; L: left; Hemi: hemisphere
